# Effectiveness of a digital data gathering system to manage the first pandemic wave among healthcare workers in a main European coronavirus disease 2019 (COVID-19) tertiary-care hospital

**DOI:** 10.1017/ash.2022.48

**Published:** 2022-04-20

**Authors:** Emanuele Sansone, Emma Sala, Elisa Albini, Mara Tiraboschi, Lorenzo Cipriani, Giuseppe De Palma

**Affiliations:** 1 Department of Medical and Surgical Specialties, Radiological Sciences, and Public Health, Unit of Occupational Health and Industrial Hygiene, University of Brescia, Brescia, Italy; 2 Unit of Occupational Health, Hygiene, Toxicology and Prevention, University Hospital “Spedali Civili Di Brescia,” Brescia, Italy

## Abstract

**Objective:**

To evaluate the information collected from workers infected with severe acute respiratory coronavirus virus 2 (SARS-CoV-2) or close contacts using a digital data gathering system (DDGS) developed at the onset of the coronavirus disease 2019 (COVID-19) pandemic to better manage the spread of infection at our hospital.

**Design:**

Observational retrospective study.

**Setting:**

Tertiary University Hospital “Spedali Civili” Hospital, Brescia, Italy.

**Participants:**

Workers (most of whom are healthcare workers) employed at the hospital.

**Methods:**

The information collected by the DDGS was transferred to the IBM SPSS statistical software package and then statistically analyzed.

**Results:**

Overall, ∼16% of the hospital workforce was infected by SARS-CoV-2 in the first pandemic wave. Nurses were the professional category with the highest infection rate (∼15%). The asymptomatic rate of infection was between 31% and 62%. Positive molecular swabs were significantly more frequent in workers undergoing the test after sending a signaling form to our DDGS. Among workers sending the signaling forms, the information about symptoms was more predictive in terms of risk, compared to the close-contact information. The concordance between molecular swabs and subsequent serological testing was significantly higher in workers signaling their at-risk condition through the DDGS.

**Conclusions:**

Overall, our data demonstrate the advantages of a digital system to gather information from workers, which is useful for managing emergencies such as the COVID-19 pandemic. This holds particularly true for large organizations such as hospitals.

At the end of 2019, a cluster of atypical pneumonia was apparent in the municipality of Wuhan, China, and was subsequently linked to a novel coronavirus, severe acute respiratory coronavirus virus 2 (SARS-CoV-2).^1^ On January 30, 2020, the World Health Organization (WHO) declared the Public Health Emergency of International Concern (PHEIC) for the spread of SARS-CoV-2 from the People’s Republic of China to 20 other countries.^2^ After the detection, on February 21, 2020, of the first autochthonous confirmed case of coronavirus disease 2019 (COVID-19) in Italy, the virus spread all over the population, particularly in the northern areas of the country. Despite the restrictive measures issued to tackle the effects of the outbreak,^3^ Italy was the Western country most severely hit, with an excess of total mortality >45,000 deaths during the first pandemic wave.^4^ The increasing number of infections forced the Italian government to enact a nationwide lockdown, which started on March 9, 2020.^5^ Two days later, the WHO declared COVID-19 a pandemic.^6^ The clinical presentation of SARS-CoV-2 infection, named COVID-19, varies widely, ranging from mild symptoms to severe illness.^7^ Asymptomatic cases of infection, which can cause silent viral transmission and therefore can sustain viral spread, have also been reported.^8^

Healthcare workers (HCWs) were significantly exposed to the risk of contagion,^9^ especially in the first pandemic wave. Infected HCWs themselves can undermine the tightness of healthcare systems^10^ and favor the spread of SARS-CoV-2 in the hospitalized population as well as the general population. Thus, the containment of infection spreading in the former has a crucial role in tackling the pandemic. Since the pandemic began, we have carried out an intense activity in contact tracing and management of SARS-CoV-2–positive HCWs, following the national and regional legislative directives.

Herein, we present the results of our clinical–epidemiologic surveillance system on HCWs, which was rapidly developed by the Occupational Health Unit (OHU) at the tertiary-care Hospital Spedali Civili of Brescia, one of the main COVID-19 hospitals in Europe. The province of Brescia, a northern Italy town, was severely hit by the pandemic, with an excess of ∼41% of deaths in 2020 compared to the average of the previous 5 years (16,608 vs 11,808).^11^ The information collected from COVID-19 cases among HCWs and their contacts, combined with the results of diagnostic testing (molecular swabs and serological tests), allowed us to document the course of pandemics in our hospital, to gauge the impact of SARS-CoV-2 on the workforce, to identify groups at higher risk among HCWs, and to assess the efficacy of the surveillance system developed and implemented.

## Methods

### Study participants

This observational retrospective study was based on data from epidemiological and clinical surveillance of workers of the tertiary-care, university hospital ASST Spedali Civili of Brescia during the first Italian phase of the SARS-CoV-2 pandemic from March 2 to May 21, 2020. According to national and regional directives, “close contact” was defined as a person who had face-to-face contact or who spent at least 15 minutes in an indoor environment with a COVID-19 patient closer than 2 m without wearing personal protective equipment (PPE; gloves, surgical mask, etc). All close contacts underwent rhino-pharyngeal molecular swab testing (RPMS). Symptomatic close contacts were quarantined at home pending the RPMS result, and asymptomatic contacts remained at work. The close contacts with negative RPMS results continued to work monitoring their symptoms for at least 2 weeks and underwent further RPMS in the middle and at the end of the clinical monitoring period. SARS-CoV-2–infected workers were isolated at home or were hospitalized in relation to the severity of their illness. SARS-CoV-2–positive workers were considered virus free after the resolution of respiratory symptoms and negative control RPMS for SARS-CoV-2. According to directives of Italian Minister of Health, until October 2020, 2 consecutive negative RPMSs were required to define recovery, whereas after October 2020, a single negative RPMS was needed.

Due to the rapid spread of infection, physicians, and trainees of the OHU adopted a digital data gathering system (DDGS) based on a digital form to collect critical clinical and epidemiological information from hospital workers. All workers were informed about the availability in the hospital intranet of digital links giving access to signaling forms that had to be compiled in each of the following cases: (1) in the event of symptoms suggestive of COVID-19; (2) when aware of a close contact (as described above) in the previous 14 days with a COVID-19 confirmed case (epidemiological form); and (3) in the event of a SARS-CoV-2–positive case among patients or personnel in a department. In the latter case, the department coordinator encouraged workers to fill in the forms. Such forms required basic sociodemographic, occupational (job, department) and clinical information, as well as details about previous contacts with confirmed COVID-19 cases, at or out of work. After compilation, each form resulted in a row on an electronic spreadsheet. SARS-CoV-2 RPMS was performed to confirm the suspect according to the appearance of COVID-19–related symptoms and/or any close contact(s) with confirmed COVID-19 case(s) in the previous 14 days. RPMS could also be prescribed in the event of COVID-19 in-ward breakouts, as well as for periodic screening in high-risk departments or following positive serological tests. RPMS tests resulting from such latter cases were excluded from analysis, whereas those resulting from screenings were compared with those performed on HCWs, accessing our DDGS to verify its effectiveness. HCWs from at-risk wards who had completed the signaling form during that same period of screening were not excluded in the analysis.

Information about the clinical course of signaling workers and confirmed COVID-19 cases was acquired through 2 different monitoring forms and was stored in 2 different databases. Whenever needed, telephonic interviews completed the information acquired by digital forms.

In the considered period, the hospital had 8,093 workers: 2,172 men, 5,921 women; mean age, 45 years (SD, 11). No significant differences were detected between sexes (Mann-Withney *P* = .344). Employees included pharmacists, nurses, obstetricians, doctors, manual workers (eg, carpenters, plumbers, electricians, drivers), psychologists, speech pathologists, social assistants, and other HCWs. The job profiles were aggregated into 7 groups according to the theoretical risk of SARS-CoV-2 infection related to specific job tasks. Table 1 shows the distributions of workers by job and sex.

**Table 1.** Distribution of the Hospital Workforce Stratified by Sex and Job Title

Job Title	Overall	Men		Women	
	No.	No.	%	No.	%
Administrative and pharmacists	824	182	22	642	78
Assistant personnel	1,300	225	17	1,075	83
Nurses	2,717	491	18	2,226	82
Doctors	2,067	921	45	1,146	55
Manual workers	289	178	62	111	38
Psychologists, speech-language pathologists, and social assistants	112	15	13	97	87
Other HCWs	784	160	20	624	80
HCWs only^a^	6,980	1,812	26	5,168	74
Total	8,093	2,172	27	5,921	73

Note. HCW, healthcare worker.

a Excluding administrative, pharmacists, and technical operators.

The study followed the Helsinki Principles; enrollment of workers and clinical and diagnostic tests were performed in the context of mandatory health surveillance according to the Italian Legislative Decree 81/2008 and emergency laws.

### Molecular and serological tests

The diagnosis of SARS-CoV-2 infection was performed at the Department of Virology of the hospital on RPMS collected and processed according to the guidelines of the Italian National Institute of Health (ISS).^12^ The specimens, preserved in UTM viral transport medium, were analyzed using reverse-transcription polymerase chain reaction (RT-PCR) to detect SARS-CoV-2 RNA sequences. As soon as serological tests became available (by the end of April 2020), screening of all hospital personnel was carried out. At that time, only a SARS-CoV-2 S1/S2 total Ig chemiluminescent assay (CLIA; Diasorin Liaison, Wein, Austria) was available. The test was performed on serum collected by venipuncture in a S-Monovette vial (Sarstedt, Newton, NC).

### Statistical analysis

The database was formatted using Microsoft Excel software (Microsoft, Redmond, WA) and was later imported into SPSS version 26.0.1 software (IBM, Chicago, IL). The normality of distributions was assessed using the Kolmogorov–Smirnov test. Categorical variables are presented as frequencies and were compared using the χ^2^ test or the Fisher exact test, as appropriate. The associations between such variables were calculated as odds ratios (ORs) and 95% confidence intervals (CIs), whereas concordance between variables was calculated using the κ statistic. Continuous variables are presented as the means ±SD (in case of a normal distribution) or medians and interquartile range (IQR) in case of a skewed distribution. A 2-sided α level of 0.05 was used for all tests.

## Results

**Fig. 1.**
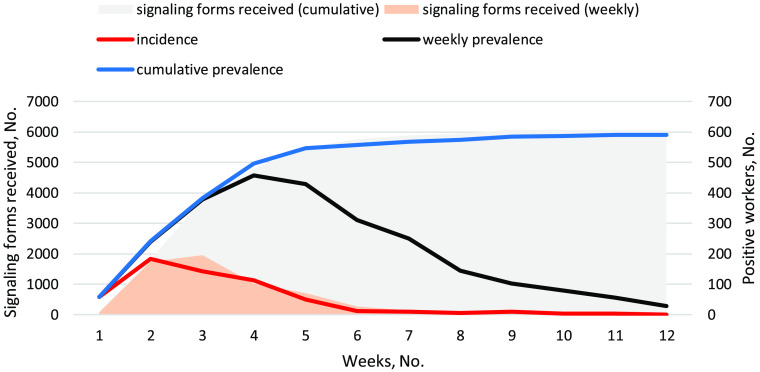
Temporal trends of SARS-CoV-2 infections (right axis and lines) and signaling activity (areas) from workers.

Figure 1 shows the weekly and cumulative incidences and prevalences of SARS-CoV-2 infection (lines, right axis) in the investigated period. The prevalence peak (N = 457) was reached in the fourth week of the outbreak, with 457 positive HCWs. At the end of the study period, 28 people were positive. The figure also shows (areas, left axis) the number of weekly and cumulative signaling forms that were sent to our unit from 3,740 workers, almost entirely during the first 6 weeks of the pandemic wave. The total number of signaling forms is about an order of magnitude higher compared to positive molecular swabs. Overall, 5,865 RPMS were performed on 3,634 workers; 590 (10% of swabs; 16% of tested workers) were positive for SARS-CoV-2. Workers sending at least one signaling form numbered 1,688, 334 (19.7%) of whom were positive for SARS-CoV-2. On July 18, 2020, 1,031 (14%) of 7,261 workers showed a positive anti–SARS-CoV-2 Ig titer. Among them, 475 (46%) also showed a positive RPMS. Of 559 workers with a previous positive RPMS, 89 (16%) were negative at a subsequent serological test, and the result was not influenced by the time interval elapsed between tests (Mann–Whitney *P* = .102). Overall, 7,349 workers were tested at least once by molecular swab or serological test. Also, 1,146 workers (16%) showed at least 1 positive RPMS or serological test. Among these 1,146 workers, SARS-CoV-2 infection was asymptomatic in 354 cases, leading to a minimum rate of asymptomatic infection (31%), which increased to 62% when workers not sending any form (n = 713) were included. For workers with at least 1 positive RPMS, we estimated a median time-to-return to work of ∼26 days (range, 6–89), which was not significantly affected by sex or age (data not shown).

Table 2 shows the distribution of the results of RPMS and serological tests in workers stratified by job title. Only for serological tests was Pearson χ^2^ test significant (*P* = .004). Looking at adapted residuals, the positive rate was significantly higher than expected in nurses and was lower in administrative and technical operators.

**Table 2.** Distributions of Molecular Swabs and Serological Tests in Workers Classified By Job Title

Job Title	Molecular Swabs		Anti-SARS-CoV-2 Igs	
	Positive/Tested, n/N (%)	Overall %	Positive/Tested, n/N (%)	Overall %
Administrative (n = 824)	33/187 (18)	4	75/701 (11)**	9
Assistant personnel (n = 1,300)	96/693 (14)	7	181/1,196 (15)	14
Nurses (n = 2,717)	235/1,477 (16)	9	396/2,526 (16)**	15
Physicians (n = 2,067)	154/867 (18)	7	245/1,731 (14)	12
Technical operators (n = 289)	9/59 (15)	3	26/268 (10)**	9
Psychologists, speech pathologists, social assistants (n = 112)	5/35 (14)	4	10/102 (10)	9
Other HCWs (n = 784)	58/316 (18)	7	98/737 (13)	13
HCWs only (n = 6,980)^a^	548/3,388 (16)	8	930/6,292 (13)	13
Total (n = 8,093)	590/3,634 (16)	7	1031/7,261 (14)	13

Note. HCW, healthcare worker.

a Excluding employees, pharmacists, and technical operators.

** P<.005.

Table 3 shows the prevalence of positive RPMS in workers stratified by job title and having compiled a signaling form or not. The probability of a positive RPMS was approximately double, on average, after signaling compared to RPMS performed for any other reason.

**Table 3.** Distribution of Molecular Swabs (RPMS) Classified by Source of Prescription in Workers Stratified by Job Title

Job Title	RPMS, Positive/Negative, (%)		Odds Ratio (95% CI)
	After a Signaling Form	For Any Other Reasons	
Administratives (n = 824)	19/90 (21)	14/128 (11)	2.2 (1.02–4.6)*
Assistant personnel (n = 1,300)	61/325 (19)	35/509 (7)	3.1 (2.0–4.9)***
Nurses (n = 2,717)	137/729 (19)	98/1,080 (9)	2.3 (1.8–3.1)***
Physicians (n = 2,067)	68/312 (22)	86/697 (12)	2.0 (1.4–2.8)***
Technical operators (n = 289)	6/28 (21)	3/40 (8)	3.4 (0.8–14.8)
Psychologists, speech-language pathologists and social assistants (n = 112)	5/29 (17)	0/15	…
Other HCWs (n = 784)	38/175 (22)	20/214 (9)	2.7 (1.5–4.8)**
HCWs only (n = 6,980)^a^	309/1,570 (20)	239/2,515 (10)	2.3 (1.9–2.8)***
Total (n = 8,093)	334/1,688 (20)	256/2,683 (10)	2.3 (1.9–2.8)***

Note. CI, confidence interval; HCW, healthcare worker.

a Excluding employees, pharmacists, and technical operators.

*P<.05, **P<.01; ***P<.0001.

The evaluation of the concordance between RPMS and serological tests may allow us to assess the accuracy of molecular testing. Testing without a strategy (ie, swab for any other reason) resulted in a poor concordance value (κ = 0.156; *P* < .0001), whereas swabs prescribed using the information collected through the digital surveillance system showed a higher agreement (κ = 0.637; *P* < .0001).

Table 4 shows that workers with COVID-19–related symptoms were at a significantly higher risk of a subsequent positive RPMS than workers with close contacts who had COVID-19, who had a higher probability of a negative RPMS result. Workers with close contacts outside work were at an approximately double risk of subsequent positive RPMS compared to those reporting close contacts at work. In such an event, contact with a COVID-19 patient(s) resulted in an approximately triple risk compared to contact with colleague(s).

**Table 4.** Distribution of Molecular Swab (RPMS) Results in Workers Signaling COVID-19–like Symptoms or Contacts at Risk

Information in Signaling Forms	RPMS, No.		Odds Ratio (95% CI)
	Positive	Negative	
**Any symptom**			
Yes	332	1,291	1.2 (1.5–1.9)**
No	108	613	
**Any contact**			
Yes	341	1,650	0.5 (0.4–0.7)**
No	99	254	
**Contact**			
Outside work	16	44	1.9 (1.0–3.4)*
At work	170	883	
With patient(s)	63	176	2.7 (1.9–4.0)***
With colleague(s)	76	578	

*P < .05; **P < .01; ***P < .0001.

## Discussion

The first wave of the SARS-CoV-2 pandemic in Italy gave rise to ∼240,000 official infections, with a peak in March (113,351 cases).^11^ Due to the early appearance of the pandemic in Italy, the Public Health System was quite unprepared for the emergency; thus, many cases passed undiagnosed.

Cases were mostly located in northern Italy, particularly in Lombardy (∼40% of the total SARS-CoV-2 positive cases in Italy), with a cumulative incidence of 739 per 100,000 inhabitants.^13^ Infected HCWs in Lombardy accounted for ∼12% of the overall burden in Italy.^14^ The prevalence of positive RPMS at our hospital was slightly lower (7% vs 8.8%) than that observed in a similar university hospital in Milan, although the spread of infection during the first pandemic phase was higher in Brescia than in Milan. Such a difference could be related to the effectiveness of the DDGS adopted in our hospital.^15^ According to other studies, nurses were exposed to a higher risk of infection (9% of positive molecular swabs and 16% of positive serology).^16^

Regarding seroprevalence, a cross-sectional study was conducted among HCWs in different Lombard provinces using serological tests on samples collected from the April 1 to 26, 2020.^17^ Among 82.961 tested participants, 10,115 HCWs (12.2%) developed IgG antibodies against SARS-CoV-2. The proportion of positive HCWs varied across different provinces; HCWs working in the most affected provinces showed higher prevalence rates. The seroprevalence among HCWs ranged from 6.7% in Monza-Brianza, with 31.3 hospitalized patients per 10.000 inhabitants, to 31.3% in Bergamo, with 78.6 hospitalized patients per 10,000 inhabitants. In another university hospital in Milan, 4,055 HCWs were tested from April 12 to 27, 2020, and 309 (7.6%) were positive.^18^ The rate of asymptomatic infection was estimated to be between 31% and 62%, ∼2–4-fold greater than previously reported.^19^

Overall, in 89 cases, the infection detected through RPMS was no longer confirmed by the subsequent serological test in the same individuals. Although the specificity of RT-PCR is thought to be almost complete,^20^ such a finding led us to consider the possibility that some results were false positives.^21^

The main results of our study support the evidence that our DDGS was effective in the identification of at-risk subjects. As shown in Table 3, the prescription of RPMS following signaling forms had an almost double probability of being positive compared to RPMS performed for other reasons. Moreover, RPMS performed after a signaling form showed a highly significant concordance with subsequent serological tests in the same individual. Both findings demonstrate that the implemented signaling system showed enough sensitivity and specificity to positively affect the appropriateness of prescribed molecular swabs, with consequent resource savings. This aspect was very relevant during the first pandemic phase because swabs were not readily available. Close contact with patients resulted at higher risk than with colleagues. We believe that such result could have been affected by an underevaluation at that time of the airborne diffusion of the SARS-CoV-2 compared to droplets, thus leading to inadequate protection (surgical masks) for the airways in many cases.

In our system, signaling forms reporting COVID-19 symptoms were significantly associated with positive RPMS. Although forms signaling any at-risk contact were negatively associated with a subsequent positive RT–PCR, a deeper analysis revealed that contacts outside work were highly predictive of a subsequent infection compared to signaled contacts at work. Among the latter, contact with patients was significantly associated with a higher risk of infection compared to contact with colleagues.

In conclusion, the obtained results demonstrate the effectiveness of the DDGS in tackling the pandemic. The availability of digitalized information allowed its rapid sharing and prompt interventions in the case of outbreaks. The resulting reduction in lead time positively affected the decision-making process, and therefore played a pivotal role in pandemic containment. Emerging and re-emerging infectious diseases pose serious health threats^21^ that also, based on our experience, require the implementation of digital technology in all health surveillance networks to gain speed and efficiency.
